# Evaluating the relationship between community water and sanitation access and the global burden of antibiotic resistance: an ecological study

**DOI:** 10.1016/S2666-5247(23)00137-4

**Published:** 2023-08

**Authors:** Erica R Fuhrmeister, Abigail P Harvey, Maya L Nadimpalli, Karin Gallandat, Argaw Ambelu, Benjamin F Arnold, Joe Brown, Oliver Cumming, Ashlee M Earl, Gagandeep Kang, Samuel Kariuki, Karen Levy, Chris E Pinto Jimenez, Jenna M Swarthout, Gabriel Trueba, Pablo Tsukayama, Colin J Worby, Amy J Pickering

**Affiliations:** a Department of Civil and Environmental Engineering, University of California, Berkeley, CA, USA; b Department of Environmental and Occupational Health Sciences, School of Public Health, University of Washington, Seattle, WA, USA; c Department of Civil and Environmental Engineering, Tufts University, Medford, MA, USA; d Gangarosa Department of Environmental Health, Emory Rollins School of Public Health, Atlanta, GA, USA; e Department of Disease Control, London School of Hygiene & Tropical Medicine, London, UK; f Department of Global Health and Development, Faculty of Public Health and Policy, London School of Hygiene & Tropical Medicine, London, UK; g Water and Health Division, Ethiopian Institute of Water Resources, Addis Ababa University, Addis Ababa, Ethiopia; h Francis I Proctor Foundation, University of California, San Francisco, CA, USA; i Department of Environmental Sciences and Engineering, Gillings School of Global Public Health, University of North Carolina, Chapel Hill, NC, USA; j Infectious Disease & Microbiome Program, Broad Institute, Cambridge, MA, USA; k The Wellcome Trust Research Laboratory, Division of Gastrointestinal Sciences, Christian Medical College, Vellore, India; l Centre for Microbiology Research, Kenya Medical Research Institute, Nairobi, Kenya; m Institutito de Microbiología, Colegio de Ciencias Biológicas y Ambientales, Universidad San Francisco de Quito, Quito, Ecuador; n Laboratorio de Genómica Microbiana, Facultad de Ciencias y Filosofía, Universidad Peruana Cayetano Heredia, Lima, Peru; o Chan Zuckerberg Biohub, San Francisco, CA, USA

## Abstract

**Background:**

Antibiotic resistance is a leading cause of death, with the highest burden occurring in low-resource settings. There is little evidence on the potential for water, sanitation, and hygiene (WASH) access to reduce antibiotic resistance in humans. We aimed to determine the relationship between the burden of antibiotic resistance in humans and community access to drinking water and sanitation.

**Methods:**

In this ecological study, we linked publicly available, geospatially tagged human faecal metagenomes (from the US National Center for Biotechnology Information Sequence Read Archive) with georeferenced household survey datasets that reported access to drinking water sources and sanitation facility types. We used generalised linear models with robust SEs to estimate the relationship between the abundance of antibiotic resistance genes (ARGs) in human faecal metagenomes and community-level coverage of improved drinking water and sanitation within a defined radii of faecal metagenome coordinates.

**Findings:**

We identified 1589 metagenomes from 26 countries. The mean abundance of ARGs, in units of log_10_ ARG fragments per kilobase per million mapped reads classified as bacteria, was highest in Africa compared with Europe (p=0·014), North America (p=0·0032), and the Western Pacific (p=0·011), and second highest in South-East Asia compared with Europe (p=0·047) and North America (p=0·014). Increased access to improved water and sanitation was associated with lower ARG abundance (effect estimate –0·22, [95% CI –0·39 to –0·05]) and the association was stronger in urban (–0·32 [–0·63 to 0·00]) than in rural (–0·16 [–0·38 to 0·07]) areas.

**Interpretation:**

Although additional studies to investigate causal effects are needed, increasing access to water and sanitation could be an effective strategy to curb the proliferation of antibiotic resistance in low-income and middle-income countries.

**Funding:**

Bill & Melinda Gates Foundation.

## Introduction

Increasing rates of antibiotic resistance among bacterial pathogens are a major public health concern.^1^ Many of the most concerning resistance phenotypes currently observed in the clinical setting are conferred through antibiotic resistance genes (ARGs). Pathogenic bacteria can acquire ARGs from other bacteria through horizontal gene transfer.^2^ In the past decade, our increased capacity to cost-effectively sequence the DNA of microbial communities (ie, metagenomic sequencing) has revealed how common ARGs are among bacteria that colonise humans, animals, and environmental niches,^3^ highlighting the scale of the challenge of preventing pathogens from acquiring ARGs.

**Figure 1:**
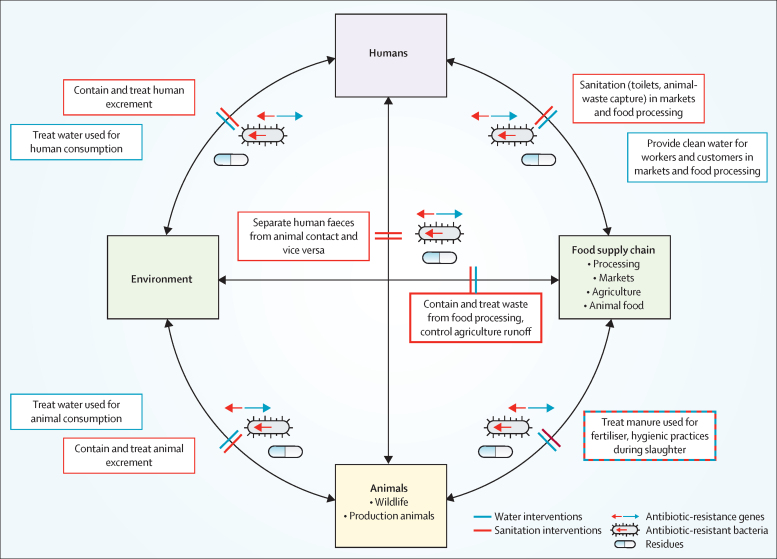
Water and Sanitation interventions needed to block the transmission of antibiotic-resistance genes, antibiotic-resistant bacteria, and the dissemination of antibiotic residues between humans, animals, the environment, and the food-supply chain

Low-income and middle-income countries (LMICs) have the highest burden of antibiotic-resistant infections,^1^ and some of the most concerning ARGs (eg, *bla*_NDM-1_ conferring carbapenem resistance and *mcr-1* conferring colistin resistance) are believed to have emerged in LMICs.^4^, ^5^ Inadequate water, sanitation, and hygiene (WASH) infrastructure and access could exacerbate the spread of resistance.^6^ Clean drinking water and flush toilets are nearly universal in most high-income countries (HICs), but availability is variable to non-existent in many LMIC settings. Access to improved WASH could prevent the emergence, transfer, and spread of antibiotic resistance in many ways.^7^ Here, we have depicted potential interventions to stop spillover between humans, animals, the environment, and food supply systems (figure 1).

Use of toilets (improved sanitation) and wastewater treatment greatly limits the load of antibiotic-resistant bacteria and ARGs that could otherwise be excreted into the environment. *Escherichia coli*, which is enriched in excreta, was implicated in 23% of total global deaths attributable to antibiotic resistance in 2019.^1^ 24 h access to clean drinking water provides an additional barrier to exposure. Furthermore, by reducing the overall infectious disease burden and accompanying need for antibiotic use, WASH services could indirectly reduce the population-level antibiotic selection mechanisms that support the propagation of resistant strains.^8^ Previous global analyses leveraging urban sewage samples have found that country-level WASH access is associated with ARG burden, lending support to this hypothesis.^9^ However, given that the majority of human faecal waste generated in LMICs does not enter centralised sewer systems,^10^ investigations that rely on urban sewage samples are not representative. Representative sampling is particularly challenging in rural settings where centralised systems are sparse.

**Research in context**

**Evidence before this study**

We searched PubMed for evidence published from database inception to Sept 20, 2020, on the effect of WASH interventions (excluding those related to animals and agriculture) on antibiotic resistance using the keyword chain: (water OR sanitation OR hygiene OR WASH) AND (antimicrobial OR antibiotic) AND resistance) NOT (“OneHealth” OR “One Health” OR animal OR livestock). No restriction was placed on language and a PubMed filter was applied to select reviews and systematic reviews (n=1420) to be screened for relevance to WASH and antibiotic resistance. We also searched the references lists of 43 selected reviews for additional relevant reviews or studies (n=16), and consulted international agency guidelines and online resources from the Joint Programming Initiative on Antimicrobial Resistance, the International Scientific Forum on Hygiene, ReAct, Resistomap, and the London School of Hygiene & Tropical Medicine Antimicrobial Resistance Centre to guide the synthesis (20 additional documents identified), for a total of 79 articles. A synthesis of wastewater treatment performance across 20 upper-middle-income and high-income countries from 1999 to 2019 reported variable removal of antibiotics (53–77%), antibiotic-resistant bacteria (99·0–99·9%) and ARGs (90·0–99·9%), broadly consistent with a qualitative systematic review of 118 studies and previous reviews showing variability by region, treatment chain, and compound. Other reviews highlighted that hand hygiene has proven effective at reducing human infections (based on ≥160 studies) and antibiotic use (based on ≥ six studies). No studies were identified on the effect of on-site sanitation systems (eg, pour-flush toilets or pit latrines), which serve an estimated 2·7 billion people globally, or faecal sludge management interventions on antibiotic resistance.

**Added value of this study**

In this study, we used 1589 publicly available human faecal metagenomes from around the world to assess the abundance of ARGs as a function of access to improved drinking water and sanitation infrastructure. This analysis provides new evidence of differences in the abundance of antibiotic resistance in the human gut across the world and finds that decreased faecal abundance of ARGs is associated with increased access to improved drinking water and sanitation.

**Implications of all the available evidence**

Current approaches to controlling antibiotic resistance in humans predominantly focus on antibiotic stewardship; however, this approach is challenging in LMICs in which infectious illnesses are generally more prevalent than in HICs and unregulated antibiotic usage is common. Along with efforts to provide other known social benefits, such as reducing infectious disease and improving gender equality, improving access to safe drinking water and sanitation could contribute to reducing the burden of antibiotic resistance. This work highlights improving access to adequate water and sanitation as a potentially effective strategy, although additional studies designed to rigorously investigate the casual relationship between WASH and antibiotic resistance are needed.

Here, we leveraged publicly available human faecal metagenomes to describe regional trends and investigate the association between community access to drinking water and sanitation and ARG abundance in the human gut. Faecal metagenome data from global populations have become increasingly available over the past decade, including from LMICs that do not have centralised water and sanitation infrastructure. The overall goal of our analysis was to examine regional differences in ARG abundance and contribute new evidence on whether inadequate WASH infrastructure might be a crucial driver of human antibiotic resistance carriage.

## Methods

### Study design and metagenome identification

In this ecological study, we obtained publicly available human faecal metagenomes, resulting from short-read (Illumina, San Diego, CA, USA) metagenomic sequencing, from the Sequence Read Archive (appendix pp 5, 6) in January, 2021. We included all available metagenomes from low-income, lower-middle income, and upper-middle income countries, as defined by World Bank income classification, and a subset of metagenomes from HICs. Studies from HICs were selected to span varying human ages and geographical locations. Longitude and latitude coordinates were obtained from the Sequence Read Archive metagenome metadata and verified with the corresponding research article. If coordinates were unavailable in the Sequence Read Archives, location was determined on the basis of the description in the research article. Additional human faecal metagenomes were contributed from Mozambique,^11^ Bangladesh,^12^ and Kenya (unpublished) by authors of these studies (JB and AJP). The Mozambique study protocol was approved by the Comité Nacional de Bioética para a Saúde (CNBS), Ministério da Saúde (333/CNBS/14), the Ethics Committee of the London School of Tropical Medicine and Hygiene (reference 8345), and the Institutional Review Board of the Georgia Institute of Technology (protocol H15160). The associated MapSan trial has been registered at ClinicalTrials.gov (NCT02362932). The Kenya study protocol was approved by the Committee for the Protection of Human Subjects at the University of California, Berkeley (protocol number 2011–09–3654), the institutional review board at Stanford University (IRB-23310), and the scientific and ethics review unit at the Kenya Medical Research Institute (protocol number SSC-2271). The Bangladesh study protocol for the original trial was approved by the International Centre for Diarrhoeal Diseases Research, Bangladesh scientific and ethical review committees (protocol number 14022) and the Stanford University human subjects institutional review board (protocol number 30456). Metagenomes were excluded if location and combined access to both drinking water and sanitation could not be determined.

### ARG identification and normalisation

ARGs were identified by mapping reads to the Comprehensive Antibiotic Resistance Database (CARD; version 3.0.9)^13^ of ARG protein sequences using BLASTx in DIAMOND (version 0.9.30.131).^14^ Results were filtered using an alignment cutoff of 25 amino acids and an identity of 95%. Paired-end reads were mapped separately, and duplicate mappings were removed. CARD entries were clustered at 80%, producing 880 antibiotic resistance gene (ARG) clusters used for naming. To normalise for total bacteria in each sample, fragments per kilobase per million mapped reads (FPKM) of ARGs were calculated using the total number of reads classified as bacteria (using Bayesian re-estimation of abundance with Kraken [Bracken]^15^) for total reads.

### Taxonomy

We used Kraken 2,^16^ with the standard database, for taxonomic classification of short reads. We used counts corrected by use of Bracken^15^ to calculate the total number of reads classified as bacteria and the relative abundance of the five most abundant families among reads classified as bacteria.

### Survey methods

Household variables, including access to improved drinking water and sanitation, were primarily obtained from geospatially tagged and nationally representative household survey datasets. US Agency for International Development Demographic and Health Surveys were obtained for the year closest to the study date (range of dates that we used in our analysis: 2010–18). If Demographic and Health Surveys data were not available (ie, for El Salvador, Mongolia, and Mexico), we obtained UNICEF Multiple Indicator Cluster Surveys from the closest available year to the study date (appendix pp 3–4). For the countries without data from either of these two sources (ie, Ecuador and China), we obtained comparable country-level or study-specific survey data (appendix pp 3–4). If the study year was unknown (n=9), we used the most recent survey available.

For countries with Demographic and Health Surveys data (which include GPS coordinates for survey clusters), survey clusters located within a 25 km radius of the metagenome coordinates were selected. Two additional radii (50 km and 75 km) were used to do a sensitivity analysis of radius threshold. For countries without Demographic and Health Surveys data (which contain information on the region or district), we determined the locally defined administrative area containing the metagenome coordinate and filtered to households within that administrative area. Within the defined geographic region (radius or administrative area), we selected urban, rural, or both types of household data, with type based on the study authors’ description of the study site. From survey data joined to each metagenome dataset, we calculated our exposures of interest: the percentage of households in the geographically defined radius with access to improved sanitation (ie, separates excreta from human contact), access to improved drinking water (ie, design protects source from contamination), and access to both improved sanitation and improved drinking water (definitions in appendix p 5). We were unable to investigate access to safely managed sanitation and drinking water, as defined by the UN Sustainable Development Goals,^17^ owing to the absence of estimates for most LMICs in our analysis. Because access to improved drinking water and sanitation could not be independently determined for HICs, we assigned a value of 99% for combined access. Additional data obtained included antibiotic consumption estimates (defined daily doses [DDDs] per 1000 people per day),^18^ gross domestic product (GDP) per capita,^19^ income classification per country as determined by World Bank designation in 2021, population density,^20^ and animal antibiotic consumption in 2010.^21^ For countries with household asset survey data available, we determined the percentage of households in the geographically defined radius owning livestock and other assets.

### Statistical analysis

We used generalised linear models to model log_10_-transformed, normalised abundance of ARGs (FPKM with total reads classified as bacteria) in total, by drug class, and individually, as a function of local access to both improved sanitation and improved drinking water in R (version 4.0.5). In separate analyses, total abundance was modelled as a function of local access to improved drinking water and improved sanitation individually. Robust SEs were used to account for clustered data (multiple samples in one georeferenced cluster). In our primary analysis, geographical region (WHO regions with North America separated from South and Central America together) and population density were included as covariates. We did two adjusted analyses. First, we included additional covariates that were available for the full set of metagenomes (antibiotic usage in humans, GDP per capita, library layout-paired or paired or single end sequencing, and rounded read length). We did a second adjusted analysis for the subset of samples with additional data available (shared sanitation, walk time to water source, animal antibiotic consumption, livestock ownership of any animal, age, sex, finished walls, floors, roof, electricity access, watch or clock, radio, TV, mobile phone, refrigerator, bike, motorcycle, car or truck, and clean fuel). The association between each covariate and outcome variable was assessed; those with a p value <0·2 were screened for collinearity using variance inflation factors. Collinear variables were iteratively eliminated until all variance inflation factors were less than 4. We also used generalised linear models with robust SEs to compare the abundance of ARGs and log_10_-transformed relative abundance of Enterobacteriaceae by region. Our prespecified analysis plan is available on the Open Science Framework (https://osf.io/n4z7f/).

Analyses of associations between abundance of ARGs and access to improved water and sanitation were done in subsets of the data separated by age (0–18 years *vs* >18 years), sex (male *vs* female), World Bank income classification (low or lower-middle *vs* upper-middle or high), geographical region, animal antibiotic usage (<50 *vs* ≥50 mg/population-corrected unit), urbanicity (rural *vs* urban), relative abundance of Enterobacteriaceae (<2% *vs* ≥2%), and human antibiotic usage (<12 *vs* ≥12 DDD per 1000 people per day).

For models of individual gene abundance, genes detected in fewer than 5% of samples were excluded and p values were corrected for multiple comparisons using the Bonferroni method.

### Role of the funding source

The funder of the study had no role in study design, data collection, data analysis, data interpretation, or writing of the report.

## Results

**Figure 2:**
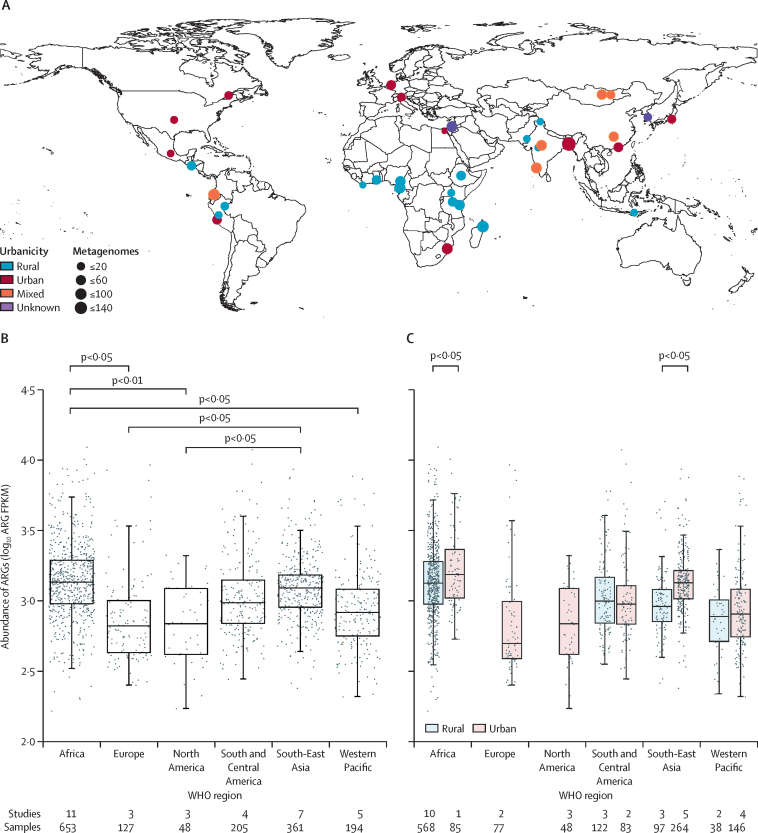
Mean abundance of antibiotic resistance genes in human fecal metagenomes from 25 countries Locations of studies included in the analysis (A; for study details see appendix pp 5–6); abundance of mapped reads classified as bacteria, by region (B); and abundance in rural and urban areas, by region (C). Dots indicate abundance, boxes indicate the 25th percentile, median, and 75th percentile, and whiskers extend to, at most, 1·5 times the IQR. P values were calculated using general linearised models with robust SEs. Eastern Mediterranean is not shown in panels B and C owing to the availability of only 1 metagenome (Egypt). Rural Europe and North America are not shown in panel C, as no metagenomes were identified from rural Europe or North America. ARGs=antibiotic resistance genes. FPKM=fragments per kilobase per million mapped reads classified as bacteria.

We identified 36 studies in total: 25 studies containing metagenomes from LMICs, eight studies from HICs, and three additional studies with metagenomes from LMICs from authors of this work. Three studies (two on China and one on Fiji) were excluded owing to an absence of data on both improved drinking water and sanitation. One study (on Brazil) was excluded owing to a high relative abundance of non-bacterial taxa and no detectable ARGs in several metagenomes. In total, 1589 metagenomes from 26 countries that were high-income (n=8), lower-middle income (n=8), upper-middle income (n=6), or low-income (n=4) were included in the analysis (figure 2; appendix pp 5–6).

The most abundant bacterial families among all metagenomes were Prevotellaceae, Bacteroidaceae, Oscillospiraceae (commonly referred to as Ruminococcaceae), Lachnospiraceae, and Enterobacteriaceae (appendix p 6). On average, the most abundant family in Africa and South-East Asia was Prevotellaceae, whereas Europe and the Western Pacific were dominated by Bacteroidaceae. There was a high degree of variability in metagenomes in the Americas, with Prevotellaceae most abundant in El Salvador and Ecuador, Bacteroidaceae most abundant in Canada, and Oscillospiraceae most abundant in Peru. Since Enterobacteriaceae are known to harbour a disproportionately high number of ARGs and ARG databases are dominated by Enterobacteriaceae alleles, we were interested in the variation in relative abundance across geographical regions. The log_10_-transformed mean relative abundance of Enterobacteriaceae, ranging from –0·03 (SD 0·7) in Europe to 0·5 (0·6) in Africa, was higher in Africa than in Europe (generalised linear model [GLM] with robust SEs p=0·010), North America (p<0·0001), and Western Pacific (p=0·010) regions. Notably, the mean relative abundance of Enterobacteriaceae was greater than 15% in Mozambique, Madagascar, Italy, Indonesia, and in one Bangladesh study.

**Figure 3:**
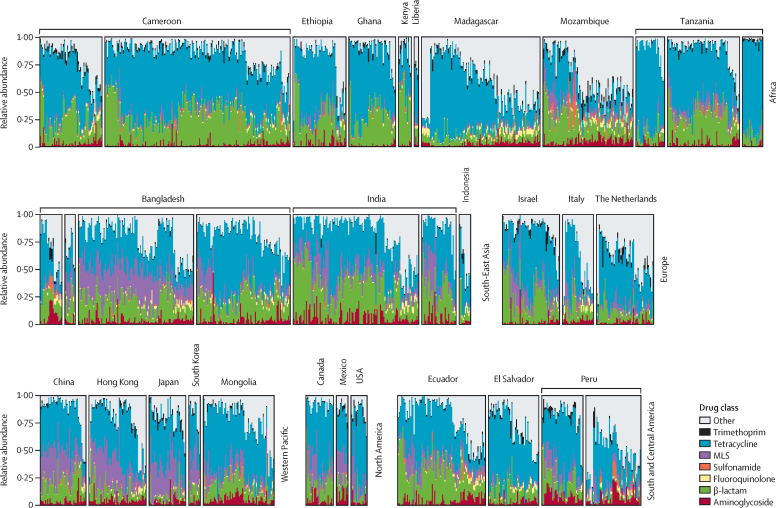
Relative abundance of antibiotic resistance genes between regions, by drug class Metagenomes are ordered by hierarchal clusters of relative abundance by drug class profiles, grouped by study and region. Each bar represents the relative proportion of each drug class in one metagenome. Eastern Mediterranean is not shown owing to the availability of only one metagenome (Egypt). Multiple studies were done in Cameroon, Tanzania, Bangladesh, India, and Peru. MLS=macrolide, lincosamide, and streptogramin.

The mean log_10_-transformed, normalised abundance of ARGs was highest in Africa compared with Europe (GLM with robust SEs p=0·014), North America (p=0·0032), and the Western Pacific (p=0·011), followed by South-East Asia, which was significantly higher than in Europe (p=0·047) and North America (p=0·014). The mean abundance of ARGs (not log_10_-transformed) in North America (FPKM 818 [SD 488) was approximately half that of Africa (1729 [1413]). Abundance trends varied by drug class (appendix p 7). β-lactamase genes were highest in Africa and South-East Asia (appendix p 7). Tetracycline resistance was ubiquitous throughout all regions (figure 3; appendix p 8). Beyond genes conferring resistance to tetracyclines, the resistome (ie, collection of all ARGs in a metagenome) was dominated by genes encoding resistance to β-lactams in Africa, Europe, and some countries in the Americas. In South-East Asia, resistance to β-lactams and macrolides, lincosamides, and streptogramins (MLS) were dominant, whereas MLS were abundant in the Western Pacific. Among metagenomes where urbanicity could be determined, there was a higher ARG abundance in urban areas in Africa (p=0·012) and South-East Asia (p=0·017; figure 2C).

Overall, increased access to improved water and sanitation was associated with a decrease in ARG abundance (table; appendix p 8). Going from 0% to 100% in access to improved water and sanitation was associated with an estimated 0·22 reduction in ARG abundance (–0·22 [95% CI –0·39 to –0·05]; p=0·0099) adjusting for region and population density. Relaxing the geographical linkage between survey data and reported sample location (using 50 km and 75 km radii) resulted in a weaker association that was not statistically significant, whereas incorporation of additional covariates resulted in a stronger magnitude of effect (appendix p 9). Across quartiles of water and sanitation access, significantly lower ARG abundance was found in regions with 50–75% access compared with 0–25% access (–0·12 [95% CI –0·25 to 0·00]; p=0·047).

**Table:** Association between ARG abundance and combined access to improved drinking water and sanitation

		**Effect estimate (95% CI)**	**p value**	**Number of metagenomes**
Change in improved water and sanitation access (0–100%)		−0·22 (−0·39 to −0·05)	0·0099	1589
Water and sanitation combined access quartile				
	0–25%	Ref	..	523
	25–50%	−0·02 (−0·15 to 0·10)	0·72	502
	50–75%	−0·12 (−0·25 to 0·00)	0·047	171
	75–100%	−0·16 (−0·34 to 0·03)	0·094	393

Effect estimates were calculated using region and population density as covariates, with robust SEs used to account for clustering (multiple metagenomes at one georeferenced cluster). Effect estimates were generated by quartiles of improved coverage for water and sanitation, in addition to the full range of water and sanitation access. For a fully adjusted model that includes region, population density, gross domestic product per person, antibiotic use in humans, read length, and library layout, see the appendix (p 8). ARG=antibiotic resistance gene.

The magnitude of association was higher for improved sanitation access alone compared with improved drinking water alone, although not statistically significant (sanitation –0·13 [95% CI –0·31 to 0·05]; p=0·15; drinking water –0·08 [–0·28 to 0·11]; p=0·40; appendix p 9).

Considering the most common drug classes (those detected in ≥80% of samples), we found increased access to improved water and sanitation was associated with a lower abundance of ARGs conferring resistance to tetracycline (–0·35 [95% CI –0·54 to –0·15]; p=0·0004) and trimethoprim (–1·15 [–1·90 to –0·39]; p=0·0030) antibiotics (appendix p 10), whereas WASH was not associated with abundance of genes conferring resistance to β-lactams, fluoroquinolones, aminoglycosides, or MLS (p>0·05 for all).

Improved water and sanitation were associated with a greater decrease in ARG abundance in urban compared with rural areas (urban –0·32 [95% CI –0·63 to 0·00]; rural –0·16 [–0·38 to 0·07], p_interaction_=0·49 appendix p 10). In the subset of metagenomes with higher relative abundance of Enterobacteriaceae, there was a greater decrease in ARG abundance compared with metagenomes with a lower relative abundance (relative abundance ≥2% –0·29 [95% CI –0·46 to –0·12]; relative abundance <2% –0·16 [–0·32 to 0·00]; p_interaction_=0·82). Of the geographical regions investigated with variation in access to WASH, improved water and sanitation was associated with the greatest decrease in ARGs in South-East Asia (–0·28 [95% CI –0·52 to –0·03]; p=0·028).

Increased access to improved water and sanitation was associated with decreased abundance of 20 of 198 resistance genes (total number of genes present in ≥5% of samples) conferring resistance to a range of antibiotic classes (appendix p 11). Of those 20 ARGs, *tetM* (conferring resistance to tetracycline), *ermT* (MLS), *qnrS* (fluoroquinolone), and *bla*_CTX-M_ Group 2 (for β-lactamases group 2 genes see appendix p 11) have been identified as current health threats owing to their presence in human-associated environments, carriage by mobile genetic elements, and presence in the genomes of ESKAPE (*Enterococcus faecium*, *Staphylococcus aureus*, *Klebsiella pneumoniae*, *Acinobacter baumannii*, *Pseudomonas aeruginosa*, and *Enterobacter* spp) pathogens.^22^ Increased access to improved water and sanitation was not statistically significantly associated with increased abundance of any of the 198 resistance genes.

## Discussion

We found that increased access to improved water and sanitation was associated with decreased ARG abundance in human faeces. We were unable to identify any metagenomes explicitly from rural sites in HICs, making it difficult to isolate the effect of urbanicity from economy type. Improved sanitation was associated with a greater decrease in ARG abundance compared with improved drinking water alone, but the greatest decrease was observed with access to both improved drinking water and sanitation. Our results suggest that increasing access to improved water and sanitation at the community-level could be an effective strategy to reduce the proliferation of antibiotic resistance. Additional studies are needed to determine if there is a causal relationship between improving WASH and antibiotic resistance burden. Also, we identified a relatively small number of human faecal metagenomes from around the world, especially in LMICs. There is a clear need for further metagenomic data from LMICs in which the burden of infectious illnesses is highest and microbiome-based research has the potential to improve human health.

The abundance of ARGs was highest in Africa, followed by South-East Asia and South and Central America. All studies in the WHO-defined African region were from sub-Saharan Africa, and thus abundance estimates are not representative of North Africa, which benefits from better WASH conditions. Total ARGs were also highest in Africa using wastewater surveillance in urban areas,^9^, ^23^ which is notable given that most metagenomes included in our analysis from Africa were from rural settings. Tetracycline resistance was abundant in all geographical regions, consistent with a previous analysis of human faecal metagenomes from 11 countries.^24^ The widespread distribution of tetracycline resistance is not surprising, given it has been used globally for decades as a broad-spectrum antibiotic in humans and animals.^25^ Additionally, there was a reasonably high abundance of genes conferring resistance to β-lactams in Africa and South-East Asia. Traveller studies indicate that northern Africa and Asia are reservoirs of β-lactam resistance, including extended-spectrum β-lactamase-producing Enterobacteriaceae.^26^

Our findings, in line with previous literature, suggest that increasing access to improved water and sanitation services within communities is a potential strategy to combat antibiotic resistance. A metagenomic analysis of wastewater samples from 60 high-income and low-income countries found that reduced open-defecation rates, increased access to improved water sources in rural areas, and increased water and sanitation investments were associated with decreased ARG abundance.^9^ We found similar associations between ARG abundance and water and sanitation access in our analysis of human faecal metagenomes from rural and urban areas in LMICs, which are probably poorly represented in wastewater surveillance efforts, given that 91% of rural and 37% of urban populations are not connected to sewerage systems.^17^ Modelling studies have also reported associations between antibiotic resistance and WASH. In a study of 73 countries globally with antibiotic consumption data, infrastructure improvements (including but not limited to improved water and sanitation) were associated with reduced prevalence of antibiotic resistant organisms (*E coli*, *Klebsiella* spp, and *S aureus*) in clinical surveillance data.^6^

Similarly, combined access to both improved water and sanitation resulted in the greatest reduction in ARG abundance in our analysis where we captured diversity of ARGs circulating in community rather than clinical settings. A modelling-based investigation of the potential effect of varying degrees of sanitation improvements on abundance of ARGs in the environment in South-East Asia found that moving from open defecation to improved sanitation, in general, provided the greatest reduction in ARGs, with proportionally smaller reductions occurring as increasingly advanced wastewater treatment processes were added.^27^ Finally, in a country-specific, experimental analysis (in contrast to the aforementioned global or WHO regional-scale, observational studies) there was no association between WASH and ARG carriage, measured using PCR, among 120 children (age <14 months) in Maputo, Mozambique.^28^ Additional studies across diverse populations are needed to further investigate the relationship between WASH and antibiotic resistance gene abundance in human guts. The period of follow-up in future intervention studies should also consider amount of time needed to clear pre-existing antibiotic resistance genes from the human gut.

An important limitation of our work is the observational, ecological design and the potential for confounding. Although our study adjusted for geographical region in all models to account for confounding socioeconomic variables, it is possible that our estimates are an overestimate of the association between ARG abundance and water and sanitation. Other limitations include an absence of standardised surveys collecting data on the quantity and diversity of antibiotics consumed by humans and animals. Current human antibiotic consumption data sources are constrained to commercial sales data or household reported use in sick children, both of which are limited in scope. Similarly, household surveys are an imperfect measure of access to improved water and sanitation conditions. Quality of improved sources, and related performance features such as drainage, can vary substantially; data collected through efforts to characterise infrastructure as safely managed are not yet available for most countries in this analysis. Additionally, Enterobacteriaceae growth in the absence of temperature control or preservation solutions during sample collection and transport could affect our results, although most metagenomic studies tightly control sample collection, transport, and testing. In the subgroup analysis, there was a stronger association between access to improved water and sanitation and ARG abundance in metagenomes with higher relative abundance of Enterobacteriaceae. Also, identification of ARGs through alignment-based methods is known to result in false positives. In a small subset of metagenomes, we used ROCker,^29^ a modelling-based strategy, to identify class A β-lactamase genes and found 75% agreement between CARD identifications and ROCker (data not shown). Finally, use of the CARD database could overstate the abundance of Enterobacteriaceae ARGs and the presence of antibiotic-resistance genes does not necessarily confer phenotypic resistance.

Our analyses emphasise the importance of choosing an ARG normalisation method carefully. Although we chose to normalise our estimates of ARG abundance using total bacterial reads, this does not account for bacterial genome size variation among samples and could provide higher estimates if ARGs are carried on smaller genomes. Alternative approaches leveraging read mapping to bacterial single-copy core genes^30^ attempt to correct for this issue, although might not account for confounders such as plasmid copy number variability and incomplete carriage of the chosen single-copy core genes set. To understand how normalisation method choice affected our results, we performed a sensitivity analysis using MicrobeCensus (version 1.1.1).^31^ Using this alternative approach, mean ARG abundance was highest in South-East Asia and the association between ARG abundance and access to improved water and sanitation was similar in magnitude and direction (but not statistically significant) to the primary analysis results (appendix p 12). Our choice to normalise by total bacterial reads was informed by observed discrepancies when we used alternative methods to identify single core copy gene numbers, indicating substantial bias, based on what reference database was used.

Education and better regulated use of antimicrobials in humans and animals (ie, stewardship) are the main approaches to managing antibiotic resistance. Mitigating infectious diseases is also a crucial component of the WHO Global Action Plan on Antimicrobial Resistance,^32^ which recommends strategies such as vaccinations and effective WASH. However, the focus for WASH has been on health-care settings rather than on community settings. In a review of the 77 action plans on antimicrobial resistance in the WHO library, only 11 countries mention community-level WASH.^33^ Although most surveillance on antimicrobial-resistant organisms has been restricted to clinical settings in LMICs, community-acquired resistance is substantial.^34^ Incorporating WASH infrastructure in community settings into national strategies could be effective for curbing antibiotic resistance in LMICs. We found the most statistically significant reduction in ARGs was associated with access to combined improvements in water and sanitation, suggesting that comprehensive WASH access might be more effective than single interventions.

## Data sharing

Sequence Read Archives project numbers for studies used in this analysis are in the appendix (pp 5–6). Compiled data used in our analysis are available on the Open Science Framework (https://osf.io/n4z7f/).

## Declaration of interests

KL reports funding from the US National Institute of Allergy and Infectious Diseases (NIAID) during the conduct of the study. AJP and JB report funding from the Bill & Melinda Gates Foundation during the conduct of the study. All other authors declare no competing interests.
