# Supplementary material for: Evaluating the relationship between community water and sanitation access and the global burden of antibiotic resistance: an ecological study
Source: Lancet Microbe. 2023 Aug;4(8):e591–600. doi: 10.1016/S2666-5247(23)00137-4 (PMC10393780; doi:10.1016/S2666-5247(23)00137-4)
Supplement: Supplementary appendix [file mmc1.pdf]

THE LANCET
Microbe

Supplementary appendix

This appendix formed part of the original submission and has been peer reviewed. We post it as supplied by the authors.

Supplement to: Fuhrmeister ER, Harvey AP, Nadimpalli ML, et al. Evaluating the relationship between community water and sanitation access and the global burden of antibiotic resistance: an ecological study. *Lancet Microbe* 2023; published online June 30. https://doi.org/10.1016/S2666-5247(23)00137-4.

Evaluating the relationship between community water and sanitation access and the global burden of antibiotic resistance: an ecological study

Erica R. Fuhrmeister, Abigail P. Harvey, Maya L. Nadimpalli, Karin Gallandat, Argaw Ambelu, Benjamin F. Arnold, Joe Brown, Oliver Cumming, Ashlee M. Earl, Gagandeep Kang, Samuel Kariuki, Karen Levy, Chris Pinto Jimenez, Jenna M. Swarthout, Gabriel Trueba, Pablo Tsukayama, Colin J. Worby, Amy J. Pickering

Appendix

List of Tables **Page**

List of Tables	Page
Table A1: Sources of variables used in this work	3-4
Table A2: Definitions used for improved and unimproved drinking water and sanitation	5
Table A3: Study details	5-6
Table A4: GLM results with all covariates	8
Table A5: GLM results using 50km and 75km thresholds for household surveys	9
Table A6: GLM results in the subset of metagenomes with complete metadata	9
Table A7: GLM results for improved sanitation coverage, improved drinking water coverage, and antibiotic usage	9
Table A8: GLM results by drug class	10
Table A9: GLM results in subsets of the data separated by age, World Bank income classification, urbanicity, antibiotic usage in animals, antibiotic usage in humans, relative abundance of *Enterobacteriaceae*, and WHO Region	10
Table A10: GLM results by individual gene	11
Table A11: Beta-lactam genes clustered at 80% similarity	11
Table A12: GLM results using MicrobeCensus	12

List of Figures

List of Figures	Page
Figure A1: Relative abundance of the five most abundant families of bacteria	6
Figure A2: Total abundance of antibiotic resistance genes by drug class	7
Figure A3: Abundance of antibiotic resistance genes by drug class and study	8
Figure A4: Abundance of antibiotic resistance genes by alternative normalization	12

Methods

Metagenome Identification

The sequence read archives (SRA) was searched for the terms "human gut metagenomes" in the organism field and "metagenomic" in the library source in December 2019 and again in June 2020. Any metagenomes with a geographic location of a low-and middle-income country, according to the World Bank designation in 2021,(1) and short-read sequencing method (Illumina) were retained. A subset of metagenomes from high-income countries were selected to provide diversity in location and age. BioProject and BioSample ID numbers were used to identify corresponding research articles. Results were further refined to include only WGS (whole genome sequencing) assays and exclude amplicon sequencing (e.g., 16S rRNA gene). Longitude and latitude coordinates were obtained from the SRA metagenome metadata and verified with the corresponding research article. If coordinates were unavailable in the SRA, location was determined based on the description in the research article. Additional metadata, when available, were extracted from corresponding research articles including sampling year, age, sex, urban or rural location, sample collection method, DNA extraction method, and library preparation method. The identified metagenome runs were downloaded using SRA Tools (v 2.9.2).

Databases for Antibiotic Resistance Genes

The Comprehensive Antibiotic Resistance Database (CARD)(v 3.0.9)(2) was downloaded in July 2020. While reads were mapped against the full database of homolog models, the CARD entries were clustered at 80% using USEARCH (v 8.1.1861)(3) producing 880 antibiotic resistance gene (ARG) clusters used for naming.

Antibiotic Resistance Gene Identification and Normalization

Downloaded short reads were trimmed using bbmap,(4) including Illumina adapter removal. For paired-end reads, complete pairs were retained. Antibiotic resistance genes were identified by mapping reads to CARD of ARG protein sequences using blastx in DIAMOND (v 0.9.30.131).(5) Results were filtered using a cutoff of 25 amino acids and an identity of 95%. In the case of paired-end reads, forward and reverse reads were mapped separately and results combined, with duplicate mappings removed.

Taxonomy

Kraken2(6) with the standard database (complete genomes in RefSeq for the bacterial, archaeal, and viral domains and Genome Reference Consortium Human Build 38 patch release 13) for taxonomy classification.

Survey Methods

Country-level estimates of GDP were obtained from the World Bank for the year of the study.(7) When the study year was missing, we used data from 2013, as this was the mean year of data collection across all studies. Income classification per country was determined by World Bank designation in 2021.(1) Population density was obtained for the 30 arc-minute region containing the metagenome coordinates from the Center for International Earth Science Information Network at Columbia University.(8) Population density for South Korea was obtained from the World Bank. Estimates for animal antibiotic consumption in 2010 per country were obtained from Our World in Data.(9) Subgroups were determined based on empirical data distributions (antibiotic usage in humans and animals, relative abundance of *Enterobacteriaceae*) or separation by categorical variables that resulted in the most balanced sample sizes (urbanicity, age, WHO region, income classification).

Secondary ARG analysis

To estimate the impact of abundance normalization method on ARG abundance, we conducted a secondary analysis using MicrobeCensus(10) as an alternative normalization approaches. Fragments per kilobase per genome equivalents was calculated using the following formula: (fragments mapped to gene)/(gene length)/genome equivalents.

Table A1. Sources of variables used in this study.

Variable	Source	Level	Units	Countries
Access to both Improved Drinking Water and Sanitation	DHS	25 km radius	%	Bangladesh (2010, 2015), Cameroon (2010, 2015), Egypt (2010), Ethiopia (2010), Ghana (2010), India (2015), Indonesia (2010-province level), Kenya (2015), Liberia (2015), Madagascar (2015), Mozambique (2015), Tanzania (2010, 2015), Peru (2012-province level)
	MICS	Province		El Salvador (2015), Mexico (2015), Mongolia (2015)
	Country-specific surveys	Province		China (2010-Hunan)
	Assumed 99%	Country		Canada, Israel, Italy, USA, Japan, Hong Kong, S. Korea, Netherlands
	Study-specific(11)	Study		Ecuador
Toilet Shared	DHS	25 km radius	%	Bangladesh (2010, 2015), Cameroon (2010, 2015), Egypt (2010), Ethiopia (2010), Ghana (2010), India (2015), Kenya (2015), Liberia (2015), Madagascar (2015), Mozambique (2015), Tanzania (2010, 2015), Peru (2012-province level)
	MICS	Province		El Salvador (2015), Mexico (2015)
	Country-specific surveys	Province		China (2010-Hunan)
Antibiotic Consumption in Humans	Browne et al.(12)	Country	DDD per 1000 persons per day	Bangladesh, Cameroon, Canada, China, Ecuador, Egypt, El Salvador, India, Indonesia, Italy, Japan, Mexico, Netherlands, Peru, S. Korea, USA, Israel, Ethiopia, Liberia, Ghana, Kenya, Tanzania, Madagascar, Mozambique, Mongolia
	Klein et al.(13)	Country		Hong Kong
Antibiotic Consumption in Animals	Our world in data(9,14,15)	Country	mg/population corrected unit	Bangladesh, Cameroon, Canada, China, Ecuador, Egypt, Ethiopia, Ghana, India, Indonesia, Israel, Italy, Japan, Kenya, Korea, Liberia, Madagascar, Mexico, Mongolia, Mozambique, Netherlands, Peru, Tanzania, USA
GDP per Capita	World Bank	Country	Billion USD/ million people	All
Income Classification	World Bank(1)	Country	NA	All
Population Density	Center for International Earth Science Information	30-arcminute region	People/km^2	Bangladesh, Cameroon, Canada, China, Ecuador, Egypt, El Salvador, Ethiopia, Ghana, India, Indonesia, Israel, Italy, Japan, Kenya, Liberia,

	Network at Columbia University(8)			Madagascar, Mexico, Mongolia, Mozambique, Netherlands, Peru, Tanzania, USA, Hong Kong
	World Bank	Country		S. Korea
Household Assets (livestock ownership, finished floors, finished walls, finished roof, electricity access, watch, clock, radio, television, mobile phone, refrigerator, bicycle, motorcycle, car/truck, clean fuel)	DHS	25 km radius	%	Bangladesh (2010, 2015), Cameroon (2010, 2015), Egypt (2010), Ethiopia (2010), Ghana (2010), India (2015), Indonesia (2010-province level), Kenya (2015), Liberia (2015), Madagascar (2015), Mozambique (2015), Tanzania (2010, 2015), Peru (2012-province level)
	MICS	Province		El Salvador (2015), Mexico (2015)
	Country-specific surveys	Province		China (2010-Hunan)
Walk time to drinking water source	DHS	25 km radius	Mean Minutes	Bangladesh (2010, 2015), Cameroon (2010, 2015), Egypt (2010), Ethiopia (2010), Ghana (2010), India (2015), Indonesia (2010-province level), Kenya (2015), Liberia (2015), Madagascar (2015), Mozambique (2015), Tanzania (2010, 2015), Peru (2012-province level)
	MICS	Province		El Salvador (2015), Mexico (2015)
	Country-level	Province		China (2010-Hunan)
Study characteristics (host age, sex, library layout, average read length, rural vs. urban, GPS coordinates)	Sequence read archives and corresponding studies	Sample	NA	All
Relative abundance of *Enterobacteriaceae*	Kraken/Bracken (6,16)	Sample	% (Total # of reads classified as Enterobacteriaceae/total number of reads classified as bacteria)	All

Table A2. Definitions and survey reponses used for improved, unimproved, and safely managed drinking water and sanitation.(17)

	Improved	Unimproved	Safely Managed
Sanitation	Facilities which ensure hygienic separation of human excreta from human contact. Survey responses: flush/pour flush to piped sewer, septic tank or pit latrine, composting toilet, ventilated improved pit latrine, or pit latrine with slab.	Survey responses: Pit latrines without a slab or platform, hanging latrines, bucket latrines, flush to somewhere else, and flush to don't know where	Use of improved facilities that are not shared with other households and where excreta are safely disposed of in situ or removed and treated offsite.
Drinking Water	Drinking water source that by the nature of its construction adequately protects the source from outside contamination, in particular with fecal matter. Survey responses: piped household connections, public taps or standpipes, boreholes or tube wells, protected dug wells, protected springs, rainwater, tanker trucks, and bottled water.	Survey responses: Unprotected dug wells, unprotected springs, and surface water.	Improved source located on premises, available when needed, and free from microbiological and priority chemical contamination.

Table A3. SRA accession numbers, country, and number of metagenomes for studies included in this analysis.

	Study Name	SRA Project No	Country	No. Metagenomes	Citation
1	Microbiome and Worm Infection	PRJNA407815	Indonesia	10	(18)
			Liberia	4	
2	Indian Human Gut Microbiome	PRJNA397112	India	110	(19)
3	Comparison of distal gut microbiota structure and function in US and Egyptian children	PRJEB8201	Egypt	1	NA
4	Subsistence strategies and hunter gatherer microbial communities	PRJNA268964	Peru	36	(20)
5	Antibiotic resistance exchange between microbiota in resource-poor settings in Latin America	PRJNA300541	El Salvador	43	(21)
			Peru	45	
6	Metagenome sequencing of the Hadza hunter-gatherer gut microbiota	PRJNA278393	Tanzania	27	(22)
7	Genome diversity of pathogenic Escherichia coli in Ecuador	PRJNA486009	Ecuador	77	(11)
8	Stool samples metagenomes from rural communities in Madagascar	PRJNA485056	Madagascar	112	(23)
9	Metagenomic sequencing of stool samples from Ethiopian individuals	PRJNA504891	Ethiopia	50	(23)
10	Gut Microbial Succession Follows Acute Secretory Diarrhea in Humans	PRJEB9150	Bangladesh	19	(24)
11	Antibiotic Treatment Leads to Fecal Escherichia coli and Coliphage Expansion in Severely Malnourished Diarrhea Patients	SRP100895	Bangladesh	9	(25)
12	Metagenomics analysis reveals features unique to Indian distal gut microbiota	PRJNA531203	India	30	(26)
13	Gut metagenomes of rural populations in Cameroon	PRJEB27005	Cameroon	57	(27)
14	Stool samples of a cohort of individuals from Tanzania (Korogwe District)	PRJNA529400	Tanzania	68	(28)
15	Metagenomic sequencing of stool samples from Ghanaian individuals	PRJNA529124	Ghana	44	(28)
16	Seasonal Cycling in the Gut Microbiome of the Hadza Hunter-Gatherers of Tanzania	PRJNA392180	Tanzania	19	(29)
17	Predicting Vibrio cholera infection and disease severity using metagenomics in a prospective cohort study	PRJNA608678	Bangladesh	82	(30)
18	Lifestyle and the presence of helminths is associated with gut microbiome composition in Cameroonians	PRJNA547591	Cameroon	175	(31)

19	The gut microbiome of Mexican children affected by obesity	PRJNA385215	Mexico	10	(32)
20	Mongolian Metagenome	PRJNA328899	Mongolia	62	(33)
21	Recent urbanization in China is correlated with a Westernized microbiome encoding increased virulence and antibiotic resistance genes	PRJNA349463	China	40	(34)
22	Metagenomic analysis of fecal microbiome as a tool towards targeted non-invasive biomarkers for colorectal cancer	ERP012177/PRJEB 10878	Hong Kong SAR	50	(35)
23	The gut microbiome of healthy Japanese and its microbial and functional uniqueness	PRJDB3601	Japan	32	(36)
24	Subsistence strategies in traditional societies distinguish gut microbiomes	PRJNA268964	USA	14	(20)
25	KOALA cohort metagenome study	PRJEB26795	Netherlands	50	(37)
26	Stability of Gut Enterotypes in Korean Monozygotic Twins and Their Association with Biomarkers and Diet	ERP002391	S. Korea	10	(38)
27	Personalized Nutrition by Prediction of Glycemic Responses	PRJEB11532	Israel	50	(39)
28	The initial state of the human gut microbiome determines its reshaping by antibiotics	PRJEB8094	Canada	24	(40)
29	Mother-to-Infant Microbial Transmission from Different Body Sites Shapes the Developing Infant Gut Microbiome	PRJNA352475	Italy	27	(41)
30	Dhaka, Bangladesh	PRJNA706606	Bangladesh	101	(42)
31	Maputo, Mozambique	PRJNA747761	Mozambique	85	(43)
32	Rural Kenya	PRJNA768833	Kenya	12	NA

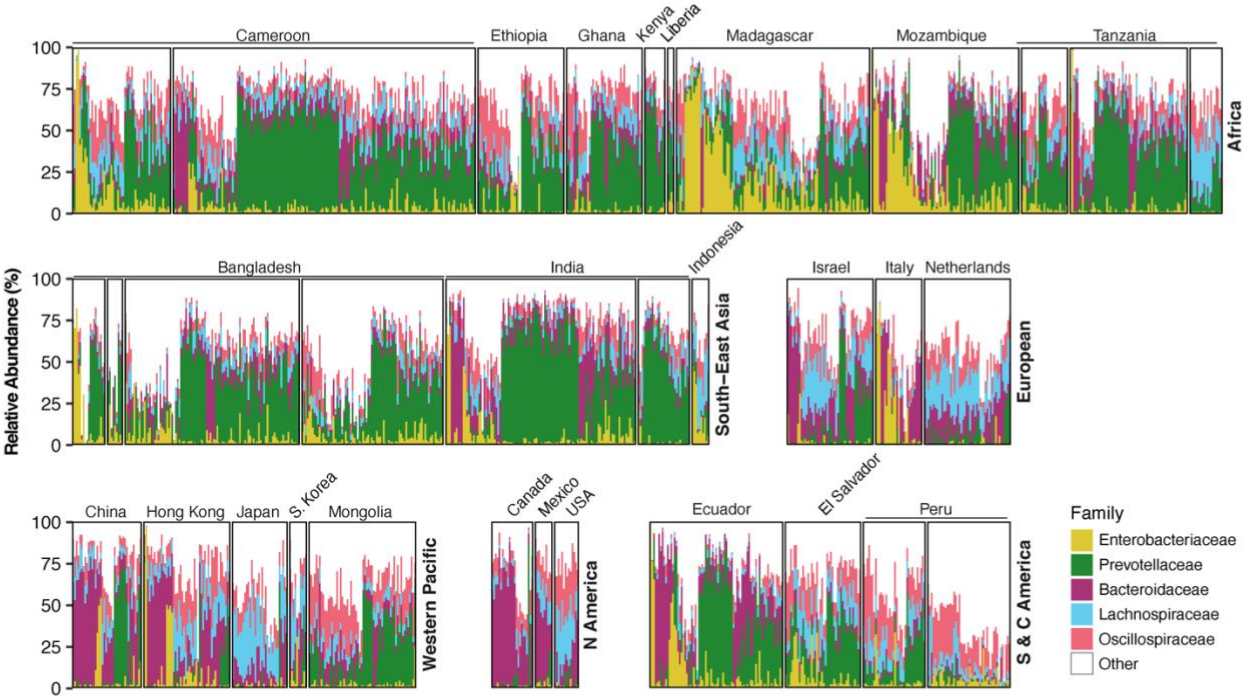

Figure A1. Relative abundance of the five most abundant families of bacteria normalized by total reads classified as bacteria. Metagenomes (each bar is one metagenome) ordered by hierarchal clustering of the family level taxa profiles and grouped by study and WHO region. Eastern Mediterranean is not shown due to the availability of only one metagenome (Egypt). Note: multiple studies conducted in Cameroon, Tanzania, Bangladesh, India, and Peru.

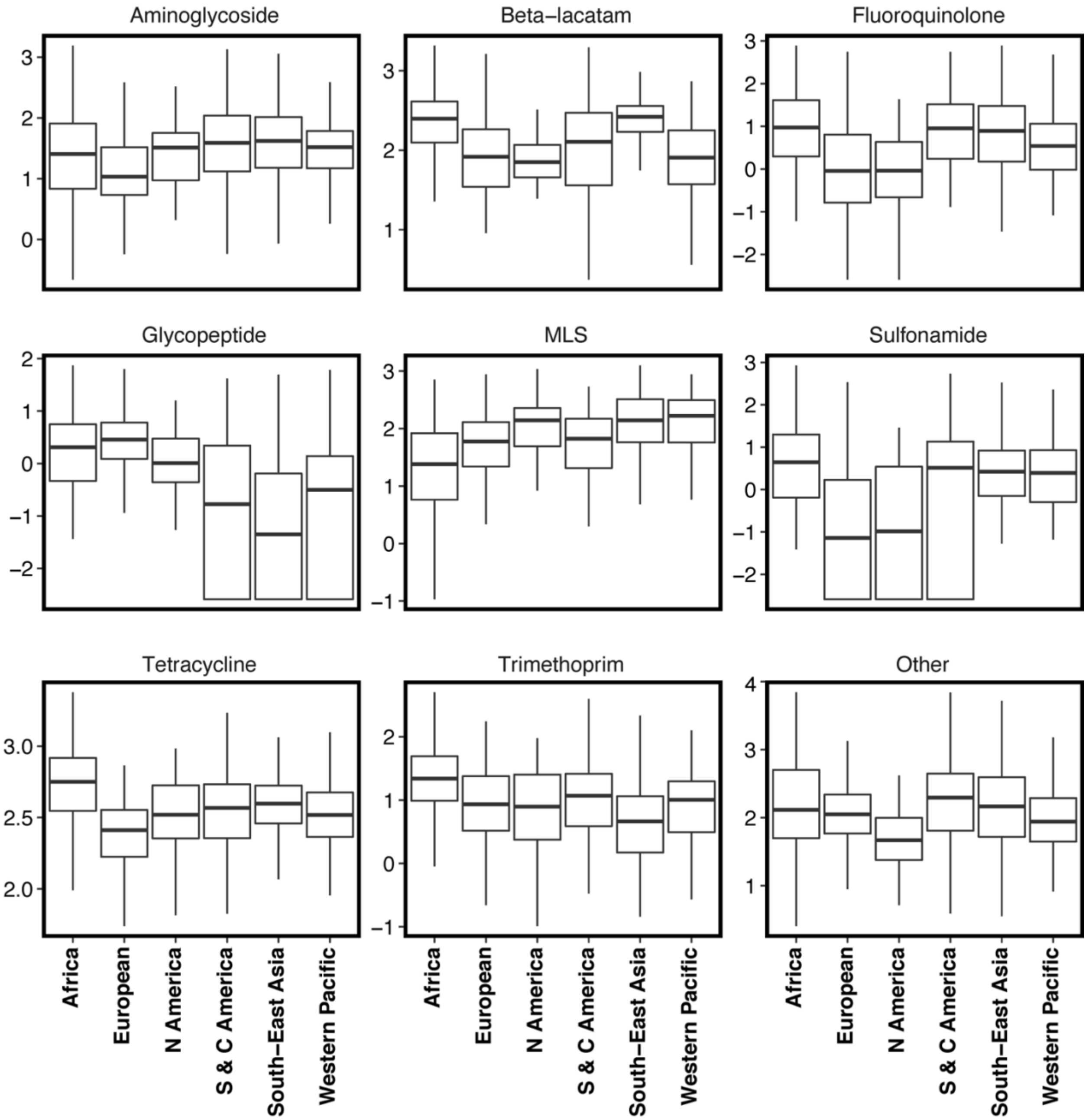

Figure A2. Total abundance of antibiotic resistance genes in units of $\log_{10}$ (RPKM ARGs using total reads classified as bacteria) by drug class and region. Boxplots consist of 25th percentile, median, 75th percentile and whiskers extend to at most 1.5x the inner quartile range. Outliers are not shown. Eastern Mediterranean is not shown due to the presence of only 1 metagenome (Egypt). MLS=Macrolide, lincosamide, streptogramin.

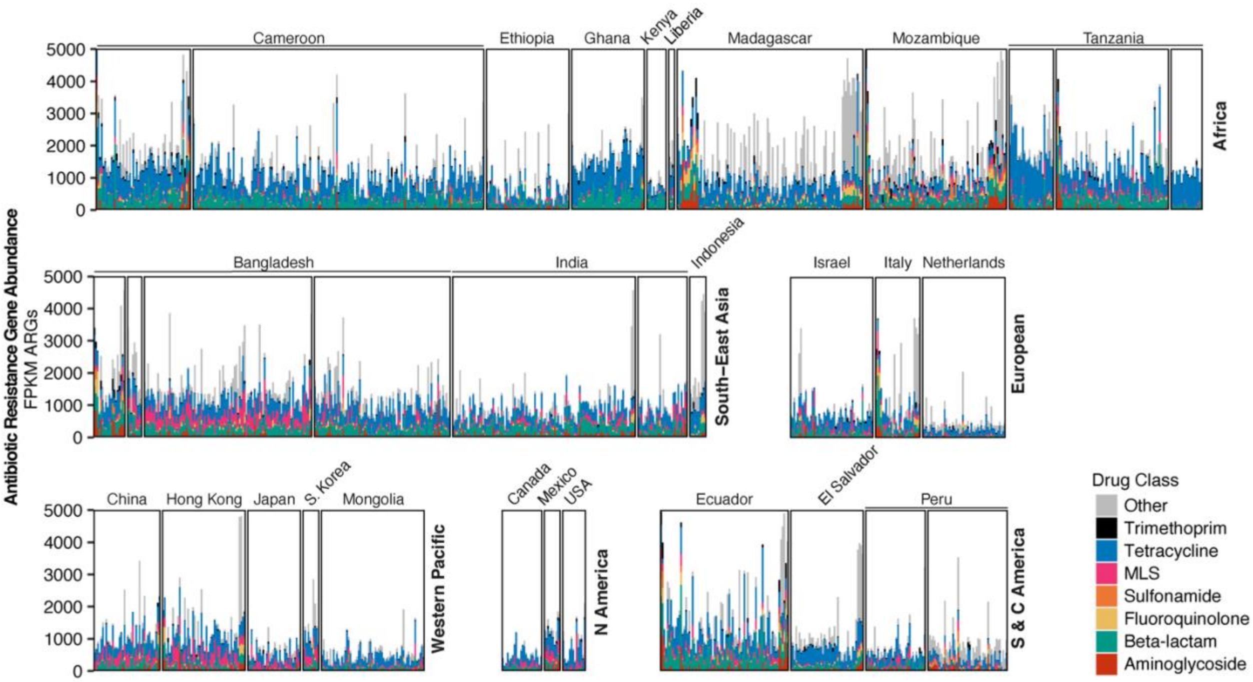

Figure A3. Abundance of antibiotic resistance genes (RPKM ARGs using total reads classified as bacteria) by drug class and study. Metagenomes were ordered by hierarchical clustering and grouped by WHO region. 13 metagenomes with abundance > 5000 were removed for visualization. Eastern Mediterranean is not shown due to the availability of only 1 metagenome (Egypt). Note: multiple studies conducted in Cameroon, Tanzania, Bangladesh, India, and Peru.

Table A4: Association between ARG abundance and combined access to both improved drinking water and sanitation adjusted for all covariates.

	**Adjusted All Covariates[1]**		
	**Effect Estimate (95% CI)**	**p-value**	**N**
	-0.19 (-0.39, 0.00)	0.049	1589
Water and Sanitation Access Quartiles			
0-25%	Reference		523
25-50%	0.06 (-0.07, 0.19)	0.39	502
50-75%	-0.04 (-0.18, 0.10)	0.55	171
75-100%	-0.09 (-0.28, 0.10)	0.33	393

[1]: Region, population density, GDP per capita, antibiotic usage in humans, read length, and library layout (Adjusted All Covariates). Additional information on covariates provided in appendix Table A2.

Table A5. General linearized model results for total antibiotic resistance gene abundance as a function of combined improved water and sanitation access using 50km and 75km thresholds for household surveys.

	Adjusted Region and Population Density		Adjusted All Covariates		
	Effect Estimate (95% CI)	p-value	Effect Estimate (95% CI)	p-value	N
**50 km**					
	-0.16 (-0.41, 0.09)	0.20	-0.12 (-0.39, 0.14)	0.37	1589
0-25%			Ref		
25-50%	-0.01 (-0.15, 0.13)	0.85	0.07 (-0.10, 0.24)	0.40	
50-75%	-0.12 (-0.25, 0.01)	0.082	-0.03 (-0.19, 0.13)	0.70	
75-100%	-0.15 (-0.35, 0.05)	0.15	-0.08 (-0.28, 0.12)	0.43	
**75 km**					
	-0.16 (-0.43, 0.11)	0.24	-0.13 (-0.42, 0.15)	0.36	1589
0-25%			Ref		
25-50%	0.00 (-0.15, 0.14)	0.95	0.06 (-0.09, 0.22)	0.43	
50-75%	-0.11 (-0.25, 0.03)	0.11	-0.04 (-0.19, 0.11)	0.58	
75-100%	-0.14 (-0.35, 0.07)	0.19	-0.08 (-0.30, 0.13)	0.45	

Table A6. General linearized model results for total antibiotic resistance gene abundance as a function of combined improved water and sanitation access in the subset of metagenomes with complete metadata.

	Adjusted Region and Population Density		Adjusted All Covariates		
	Estimate (95% CI)	p-value	Estimate (95% CI)	p-value	N
25 km	-0.15 (-0.49, 0.19)	0.39	-0.39 (-0.65, -0.13)	0.0028	961
50 km	0.25 (-0.45, 0.95)	0.48	-0.20 (-0.79, 0.38)	0.50	961
75 km	0.31 (-0.48,1.10)	0.44	-0.35 (-0.76, 0.06)	0.093	961

Table A7. General linearized model results for total antibiotic resistance gene abundance as a function of improved sanitation coverage, improved drinking water coverage, and antibiotic usage in humans. Models were conducted individually. N=1589

	Adjusted Region and Population Density		Adjusted All Covariates	
	Effect Estimate (95% CI)	p-value	Effect Estimate (95% CI)	p-value
Sanitation only	-0.13 (-0.31, 0.05)	0.15	-0.12 (-0.29, 0.05)	0.18
Drinking water only	-0.08 (-0.28, 0.11)	0.40	0.00 (-0.25, 0.24)	0.99
Antibiotic usage only	0.00 (0.00, 0.01)	0.35	0.00 (0.00, 0.01)	0.15

Table A8. General linearized model results for total antibiotic resistance gene abundance in each drug class as a function of combined improved drinking water and sanitation. Adjusted for WHO region and population density. Drug classes with ≥ 20% of samples considered non-detects are not shown. N=1589

Drug Class	Estimate (95% CI LB, UB)	p-value
Tetracycline	-0.35 (-0.54, -0.15)	0.0004
Beta-lactam	-0.27 (-0.76, 0.21)	0.27
Aminoglycoside	-0.04 (-0.44, 0.35)	0.82
MLS	-0.04 (-0.55, 0.48)	0.89
Fluoroquinolone	-0.56 (-1.29, 0.16)	0.13
Trimethoprim	-1.15 (-1.90, -0.39)	0.0030

Table A9. Association between ARG abundance and combined access to both improved drinking water and sanitation in subsets of the data separated by age, sex, World Bank income classification, urbanicity, antibiotic usage in animals, antibiotic usage in humans, relative abundance of *Enterobacteriaceae*, and geographical Region. Region included as a covariate in all models, except for the WHO region model. Population density included as a covariate for all models, except for urbanicity. Robust standard errors were used to account for clustering (multiple metagenomes at one georeferenced cluster). Interaction p-value for WHO Region determined using a Wald test. PCU: Population-corrected unit. DDD: Defined daily dose. RA: Relative abundance.

Subgroup	n	Estimate (95% CI)	p-value	Interaction p-value
**Age**				
18+ years	992	-0.16 (-0.30, -0.03)	0.018	0.73
0-18 years	555	-0.06 (-0.45, 0.34)	0.78	
**Sex**				
Female	622	-0.24 (-0.43, -0.06)	0.0093	0.72
Male	571	-0.28 (-0.46, -0.10)	0.0021	
**Income**				
Low, lower-middle	1049	-0.22 (-0.40, -0.05)	0.013	0.96
Upper-middle, high	540	-0.20 (-0.64, 0.24)	0.37	
**Rural vs Urban**				
Rural	825	-0.16 (-0.38, 0.07)	0.17	0.49
Urban	704	-0.32 (-0.63, 0.00)	0.050	
**Antibiotic Usage in Animals**				
< 50 mg/PCU	834	-0.22 (-0.42, -0.02)	0.034	0.83
≥ 50 mg/PCU	651	-0.24 (-0.70, 0.22)	0.31	
**Antibiotic Usage in Humans**				
< 12 DDD per 1000 persons per day	786	-0.11 (-0.41, 0.18)	0.46	0.56
≥ 12 DDD per 1000 persons per day	803	-0.17 (-0.37, 0.03)	0.10	
**Relative Abundance of *Enterobacteriaceae***				
RA *Enterobacteriaceae* < 2%	807	-0.16 (-0.32, 0.00)	0.056	0.82
RA *Enterobacteriaceae* ≥ 2%	782	-0.29 (-0.46, -0.12)	0.0006	
**WHO Region**				
Africa	653	-0.14 (-0.50, 0.23)	0.46	0.18
S & C America	205	-0.58 (-1.40, 0.24)	0.16	
South-East Asia	361	-0.28 (-0.52, -0.03)	0.028	
Western Pacific	194	-0.04 (-0.38, 0.29)	0.80	

Table A10. Adjusted and unadjusted general linearized model results for abundance of individual antibiotic resistance genes detected in at least 5% of metagenomes and significant at p-value <0.05 in the model adjusted for region and population density.

		Adjusted for Region and Population Density		Adjusted all covariates		Current Threat
Gene	Drug Class	Effect	P-value	Effect	p-value	
tetB(P)	tetracycline	-2.15 (-2.89, -1.41)	<0.0001	-1.99 (-2.54, -1.43)	<0.0001	
tetM	tetracycline	-2.06 (-2.81, -1.31)	<0.0001	-2.39 (-3.13, -1.65)	<0.0001	Y
qnrS	fluoroquinolone	-1.33 (-1.87, -0.79)	0.0003	-1.35 (-1.93, -0.77)	0.001	Y
patB	fluoroquinolone	-1.04 (-1.48, -0.60)	0.0008	-1.24 (-1.69, -0.79)	<0.0001	
RlmA(II)	lincosamide, macrolide	-0.78 (-1.11, -0.45)	0.0010	-0.88 (-1.26, -0.51)	0.0007	
ermQ	lincosamide, macrolide, streptogramin	-1.32 (-1.89, -0.74)	0.0013	-1.52 (-2.11, -0.93)	0.0001	
cmeB	multi drug	-0.52 (-0.74, -0.29)	0.0013	-0.48 (-0.70, -0.26)	0.0031	
C. perfringens mprF	peptide	-0.60 (-0.87, -0.33)	0.0031	-0.51 (-0.69, -0.34)	<0.0001	
DHA	beta-lactam	-0.71 (-1.03, -0.38)	0.0035	-0.77 (-1.10, -0.44)	0.0010	
pmrA	fluoroquinolone	-0.76 (-1.11, -0.41)	0.0036	-0.94 (-1.32, -0.56)	0.0002	
mef(B)	macrolide	-0.34 (-0.49, -0.18)	0.0045	-0.31 (-0.47, -0.14)	0.039	
tetA(P)	tetracycline	-1.63 (-2.43, -0.83)	0.013	-1.61 (-2.18, -1.04)	<0.0001	
msrE	multi drug	-0.54 (-0.80, -0.27)	0.013	-0.51 (-0.75, -0.27)	0.0048	
efrB	multi drug	-0.62 (-0.93, -0.31)	0.017	-0.68 (-0.97, -0.40)	0.0005	
mtrD	multi drug	-0.27 (-0.40, -0.13)	0.021	-0.30 (-0.43, -0.17)	0.0009	
patA	fluoroquinolone	-0.97 (-1.47, -0.47)	0.029	-1.14 (-1.64, -0.63)	0.0019	
ermT	lincosamide, macrolide, streptogramin	-1.41 (-2.13, -0.68)	0.031	-1.69 (-2.34, -1.04)	0.0001	Y
sul3	sulfonamide	-0.49 (-0.75, -0.24)	0.033	-0.48 (-0.74, -0.22)	0.052	
vanSC	glycopeptide	-0.50 (-0.76, -0.23)	0.040	-0.48 (-0.74, -0.21)	0.086	
CTX-M Group 2	beta-lactam	-0.77 (-1.18, -0.36)	0.040	-0.74 (-1.11, -0.37)	0.020	Y

Table A11. Genes clustered at 80% similarity for naming purposes in our analysis. Embolden genes are listed as "current threats" in Zhang et al.(44)

**Group Name**	**Genes Clustered into Group**
CTX-M Group 2	CTX-M-11, CTX-M-42, CTX-M-144, CTX-M-34, CTX-M-88, CTX-M-116, CTX-M-12, CTX-M-69, CTX-M-114, **CTX-M-15,** CTX-M-136, CTX-M-33, CTX-M-60, CTX-M-139, CTX-M-10, CTX-M-22, CTX-M-82, CTX-M-158, CTX-M-117, CTX-M-62, CTX-M-58, CTX-M-36, CTX-M-32, CTX-M-157, CTX-M-132, CTX-M-109, CTX-M-23, CTX-M-142, CTX-M-68, CTX-M-3, CTX-M-96, **CTX-M-55**, CTX-M-61, CTX-M-54, CTX-M-53, CTX-M-107, CTX-M-108, CTX-M-1, CTX-M-103, CTX-M-30, CTX-M-66, CTX-M-72, CTX-M-52, CTX-M-80, CTX-M-29, CTX-M-156, CTX-M-71, CTX-M-155, CTX-M-123, CTX-M-79, CTX-M-28, CTX-M-37, CTX-M-101

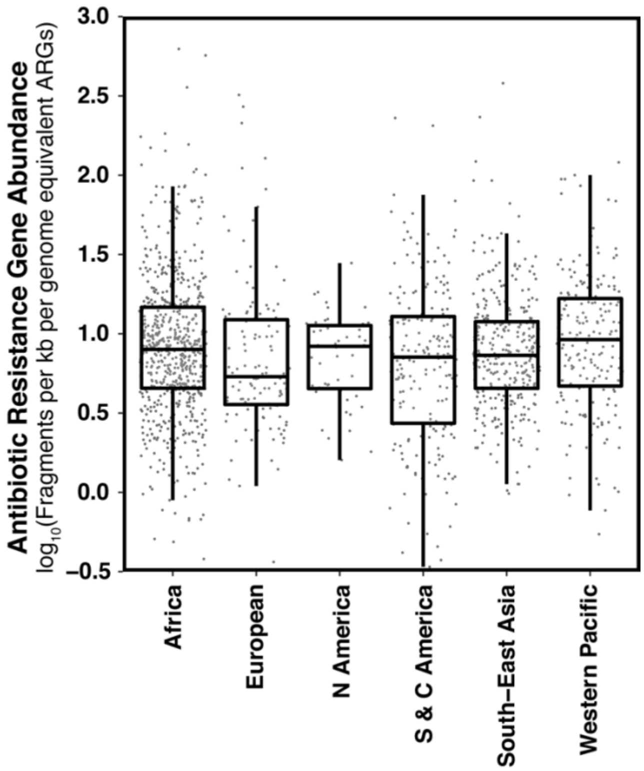

Figure A4. Abundance of antibiotic resistance genes in units of log_{10} fragments per kilobase per genome equivalent. Boxplots consist of 25th percentile, median, 75th percentile and whiskers extend to at most 1.5x the inner quartile range

Table A12. Association between ARG abundance and combined access to both improved drinking water and sanitation normalized by genome equivalents calculated using MicrobeCensus.

	Adjusted Region and Population Density		Adjusted All Covariates		
	Effect Estimate (95% CI)	p-value	Effect Estimate (95% CI)	p-value	N
	-0.24 (-0.53, 0.04)	0.095	-0.13 (-0.47,0.2)	0.42	1552
Water and Sanitation Access Quartiles					
0-25%	Reference		Reference		519
25-50%	-0.04 (-0.22, 0.14)	0.69	0.08 (-0.12, 0.29)	0.43	475
50-75%	-0.16 (-0.35, 0.04)	0.12	-0.02 (-0.25, 0.21)	0.85	167
75-100%	-0.21 (-0.46, 0.05)	0.11	-0.10 (-0.41, 0.20)	0.51	391

References

1. World Bank Country and Lending Groups [Internet]. [cited 2021 Jun 22]. Available from: https://datahelpdesk.worldbank.org/knowledgebase/articles/906519-world-bank-country-and-lending-groups

2. Alcock BP, Raphenya AR, Lau TTY, Tsang KK, Bouchard M, Edalatmand A, et al. CARD 2020: antibiotic resistome surveillance with the comprehensive antibiotic resistance database. Nucleic Acids Res. 2020 Jan 8;48(D1):D517–25.

3. Edgar RC. Search and clustering orders of magnitude faster than BLAST. Bioinformatics. 2010 Oct 1;26(19):2460–1.

4. Bushnell B. BBTools [Internet]. 2014. Available from: https://sourceforge.net/projects/bbmap/

5. Buchfink B, Xie C, Huson DH. Fast and sensitive protein alignment using DIAMOND. Nat Methods. 2015;12(1):59–60.

6. Wood DE, Lu J, Langmead B. Improved metagenomic analysis with Kraken 2. Genome Biol. 2019 Nov 28;20(1):257.

7. The World Bank. GDP (current US$) | Data [Internet]. [cited 2021 Jun 23]. Available from: https://data.worldbank.org/indicator/NY.GDP.MKTP.CD

8. Center For International Earth Science Information Network-CIESIN-Columbia University. Gridded Population of the World, Version 4 (GPWv4): Population Density, Revision 11 [Internet]. Palisades, NY: Socioeconomic Data and Applications Center (SEDAC); 2017 [cited 2021 Jun 23]. Available from: https://sedac.ciesin.columbia.edu/data/set/gpw-v4-population-density-rev11

9. Antibiotic use in livestock [Internet]. Our World in Data. [cited 2021 Jun 17]. Available from: https://ourworldindata.org/grapher/antibiotic-use-in-livestock

10. Nayfach S, Pollard KS. Average genome size estimation improves comparative metagenomics and sheds light on the functional ecology of the human microbiome. Genome Biol. 2015 Mar 25;16(1):51.

11. Peña-Gonzalez A, Soto-Girón MJ, Smith S, Sistrunk J, Montero L, Páez M, et al. Metagenomic Signatures of Gut Infections Caused by Different Escherichia coli Pathotypes. Appl Environ Microbiol. 85(24):e01820-19.

12. Browne AJ, Chipeta MG, Haines-Woodhouse G, Kumaran EPA, Hamadani BHK, Zaraa S, et al. Global antibiotic consumption and usage in humans, 2000–18: a spatial modelling study. Lancet Planet Health. 2021 Dec 1;5(12):e893–904.

13. Klein EY, Van Boeckel TP, Martinez EM, Pant S, Gandra S, Levin SA, et al. Global increase and geographic convergence in antibiotic consumption between 2000 and 2015. Proc Natl Acad Sci. 2018 Apr 10;115(15):E3463–70.

14. Van Boeckel TP, Brower C, Gilbert M, Grenfell BT, Levin SA, Robinson TP, et al. Global trends in antimicrobial use in food animals. Proc Natl Acad Sci. 2015 May 5;112(18):5649–54.

15. Veterinary Medicines Division. Sales of veterinary antimicrobial agents in 30 European countries in 2015 [Internet]. European Surveillance of Veterinary Antimicrobial Consumption; 2017. Available from: https://www.ema.europa.eu/en/documents/report/seventh-esvac-report-sales-veterinary-antimicrobial-agents-30-european-countries-2015_en.pdf

16. Lu J, Breitwieser FP, Thielen P, Salzberg SL. Bracken: estimating species abundance in metagenomics data. PeerJ Comput Sci. 2017 Jan 2;3:e104.

17. WHO and UNICEF. Progress on drinking-water, sanitation and hygiene: 2017 update and SDG baselines [Internet]. 2017 [cited 2021 Jun 23]. Available from: https://www.who.int/publications-detail-redirect/9789241512893

18. Rosa BA, Supali T, Gankpala L, Djuardi Y, Sartono E, Zhou Y, et al. Differential human gut microbiome assemblages during soil-transmitted helminth infections in Indonesia and Liberia. Microbiome. 2018 Feb 28;6(1):33.

19. Dhakan DB, Maji A, Sharma AK, Saxena R, Pulikkan J, Grace T, et al. The unique composition of Indian gut microbiome, gene catalogue, and associated fecal metabolome deciphered using multi-omics approaches. GigaScience [Internet]. 2019 Mar 1 [cited 2021 Sep 1];8(3). Available from: https://doi.org/10.1093/gigascience/giz004

20. Obregon-Tito AJ, Tito RY, Metcalf J, Sankaranarayanan K, Clemente JC, Ursell LK, et al. Subsistence strategies in traditional societies distinguish gut microbiomes. Nat Commun. 2015 Mar 25;6(1):6505.

21. Pehrsson EC, Tsukayama P, Patel S, Mejía-Bautista M, Sosa-Soto G, Navarrete KM, et al. Interconnected microbiomes and resistomes in low-income human habitats. Nature. 2016 May;533(7602):212–6.

22. Rampelli S, Schnorr SL, Consolandi C, Turroni S, Severgnini M, Peano C, et al. Metagenome Sequencing of the Hadza Hunter-Gatherer Gut Microbiota. Curr Biol. 2015 Jun 29;25(13):1682–93.

23. Pasolli E, Asnicar F, Manara S, Zolfo M, Karcher N, Armanini F, et al. Extensive Unexplored Human Microbiome Diversity Revealed by Over 150,000 Genomes from Metagenomes Spanning Age, Geography, and Lifestyle. Cell. 2019;176(3):649-662.e20.

24. David LA, Weil A, Ryan ET, Calderwood SB, Harris JB, Chowdhury F, et al. Gut Microbial Succession Follows Acute Secretory Diarrhea in Humans. mBio. 6(3):e00381-15.

25. Kieser S, Sarker SA, Berger B, Sultana S, Chisti MJ, Islam SB, et al. Antibiotic Treatment Leads to Fecal Escherichia coli and Coliphage Expansion in Severely Malnourished Diarrhea Patients. Cell Mol Gastroenterol Hepatol. 2018 Mar;5(3):458-460.e6.

26. Kaur K, Khatri I, Akhtar A, Subramanian S, Ramya TNC. Metagenomics analysis reveals features unique to Indian distal gut microbiota. PLOS ONE. 2020 Apr 8;15(4):e0231197.

27. Lokmer A, Cian A, Froment A, Gantois N, Viscogliosi E, Chabé M, et al. Use of shotgun metagenomics for the identification of protozoa in the gut microbiota of healthy individuals from worldwide populations with various industrialization levels. PLOS ONE. 2019 Feb 6;14(2):e0211139.

28. Tett A, Huang KD, Asnicar F, Fehlner-Peach H, Pasolli E, Karcher N, et al. The Prevotella copri Complex Comprises Four Distinct Clades Underrepresented in Westernized Populations. Cell Host Microbe. 2019 Nov 13;26(5):666-679.e7.

29. Smits SA, Leach J, Sonnenburg ED, Gonzalez CG, Lichtman JS, Reid G, et al. Seasonal cycling in the gut microbiome of the Hadza hunter-gatherers of Tanzania. Science. 2017 Aug 25;357(6353):802–6.

30. Levade I, Saber MM, Midani FS, Chowdhury F, Khan AI, Begum YA, et al. Predicting Vibrio cholerae Infection and Disease Severity Using Metagenomics in a Prospective Cohort Study. J Infect Dis. 2021 Jan 15;223(2):342–51.

31. Rubel MA, Abbas A, Taylor LJ, Connell A, Tanes C, Bittinger K, et al. Lifestyle and the presence of helminths is associated with gut microbiome composition in Cameroonians. Genome Biol. 2020 May 25;21(1):122.

32. Maya-Lucas O, Murugesan S, Nirmalkar K, Alcaraz LD, Hoyo-Vadillo C, Pizano-Zárate ML, et al. The gut microbiome of Mexican children affected by obesity. Anaerobe. 2019 Feb 1;55:11–23.

33. Liu W, Zhang J, Wu C, Cai S, Huang W, Chen J, et al. Unique Features of Ethnic Mongolian Gut Microbiome revealed by metagenomic analysis. Sci Rep. 2016 Oct 6;6(1):34826.

34. Winglee K, Howard AG, Sha W, Gharaibeh RZ, Liu J, Jin D, et al. Recent urbanization in China is correlated with a Westernized microbiome encoding increased virulence and antibiotic resistance genes. Microbiome. 2017 Sep 15;5(1):121.

35. Yu J, Feng Q, Wong SH, Zhang D, Liang Q yi, Qin Y, et al. Metagenomic analysis of faecal microbiome as a tool towards targeted non-invasive biomarkers for colorectal cancer. Gut. 2017 Jan 1;66(1):70–8.

36. Nishijima S, Suda W, Oshima K, Kim SW, Hirose Y, Morita H, et al. The gut microbiome of healthy Japanese and its microbial and functional uniqueness. DNA Res. 2016 Apr 1;23(2):125–33.

37. Zhong H, Penders J, Shi Z, Ren H, Cai K, Fang C, et al. Impact of early events and lifestyle on the gut microbiota and metabolic phenotypes in young school-age children. Microbiome. 2019 Jan 4;7(1):2.

38. Lim MY, Rho M, Song YM, Lee K, Sung J, Ko G. Stability of Gut Enterotypes in Korean Monozygotic Twins and Their Association with Biomarkers and Diet. Sci Rep. 2014 Dec 8;4(1):7348.

39. Zeevi D, Korem T, Zmora N, Israeli D, Rothschild D, Weinberger A, et al. Personalized Nutrition by Prediction of Glycemic Responses. Cell. 2015 Nov 19;163(5):1079–94.

40. Raymond F, Ouameur AA, Déraspe M, Iqbal N, Gingras H, Dridi B, et al. The initial state of the human gut microbiome determines its reshaping by antibiotics. ISME J. 2016 Mar;10(3):707–20.

41. Ferretti P, Pasolli E, Tett A, Asnicar F, Gorfer V, Fedi S, et al. Mother-to-Infant Microbial Transmission from Different Body Sites Shapes the Developing Infant Gut Microbiome. Cell Host Microbe. 2018 Jul 11;24(1):133-145.e5.

42. Nadimpalli ML, Lanza VF, Montealegre MC, Sultana S, Fuhrmeister ER, Worby CJ, et al. Drinking water chlorination has minor effects on the intestinal flora and resistomes of Bangladeshi children. Nat Microbiol. 2022 May;7(5):620–9.

43. Kim M, Rodriguez-R LM, Hatt JK, Kayali O, Nalá R, Dunlop AL, et al. Higher pathogen load in children from Mozambique vs. USA revealed by comparative fecal microbiome profiling. ISME Commun. 2022 Aug 18;2(1):1–10.

44. Zhang AN, Gaston JM, Dai CL, Zhao S, Poyet M, Groussin M, et al. An omics-based framework for assessing the health risk of antimicrobial resistance genes. Nat Commun. 2021 Aug 6;12(1):4765.
